# Utilization of a PFA-GGBS-Based Precursor in Geopolymer Concrete Production as a Sustainable Substitute for Conventional Concrete

**DOI:** 10.3390/ma18061309

**Published:** 2025-03-16

**Authors:** Jonathan Oti, Blessing O. Adeleke, Lito R. Casabuena, John M. Kinuthia, Samuel Sule

**Affiliations:** 1 Faculty of Computing, Engineering and Science, University of South Wales, Pontypridd CF37 1DL, UK; 2 Faculty of Engineering, Department of Civil & Environmental Engineering, University of Portharcourt, Rivers State PMB 500272, Nigeria

**Keywords:** geopolymer, GGBS, PFA, AAS, alkaline solutions, aluminosilicate materials, alkaline/precursor (A/P), water to binder (W/B), compressive strength, split tensile strength, flexural strength

## Abstract

Awareness of environmental sustainability is driving the shift from conventional Portland cement, a major contributor to carbon dioxide emissions, to more sustainable alternatives. This study focuses on developing a geopolymer concrete by optimizing geopolymer concrete mixtures with various ratios of Ground Granulated Blast Furnace Slag (GGBS) and pulverized fly ash (PFA) as precursors, aiming to find a mix that maximizes strength while minimizing environmental impacts. The precursor was activated using a laboratory-synthesized silica fume (SF)-derived sodium silicate solution in combination with NaOH at a molarity of 10M. This study aims to find the optimal geopolymer concrete mix with a 0.55 water-to-binder ratio, a 0.40 alkali-to-precursor ratio, and a 1:1 sodium silicate to sodium hydroxide ratio. Ordinary Portland cement was used as the control mix binder (C), while the geopolymer mixes included varying GGBS-PFA compositions [CL0 (50% GGBS—50% PFA), CL1 (60% GGBS—40% PFA), CL2 (70% GGBS—30% PFA), CL3 (80% GGBS—20% PFA), and CL4 (90% GGBS—10% PFA)]. The engineering performance of the mixtures was assessed using slump, unconfined compressive strength, split tensile, and flexural strength tests in accordance with their relevant standards. Observations showed that GPC specimens exhibited similar or slightly higher strength values than conventional concrete using PC. In addition to strength, geopolymers have a smaller environmental footprint, consuming less energy and reducing greenhouse gas emissions. These qualities make geopolymer concrete a sustainable construction option that aligns with global efforts to reduce carbon emissions and conserve resources.

## 1. Introduction

Concrete is the most widely used construction material globally, essential for infrastructure and residential projects. Ordinary Portland cement (OPC), its primary binder, has been a mainstay in modern construction due to its affordability, strength, and the availability of natural raw materials like limestone and clay. However, producing Portland cement is energy intensive and contributes significantly to carbon dioxide (CO₂) emissions, accounting for up to 90% of the emissions in cement production and about 8% of global CO₂ output [1,2]. Therefore, producing a sustainable construction material with a lower carbon footprint both at the manufacture and utilization stages is expedient for enhancing the durability and sustainability of concrete using supplementary cementitious materials. One of these innovations is the development of a sustainable material technology (geopolymer) by Joseph Davidovits in the 1970s, which involved an environmentally friendly binder created from the combination of raw materials and an alkaline activator [3,4,5].

Geopolymers are inorganic, aluminosilicate materials created through a chemical reaction that serve as sustainable binders in concrete production [6]. These materials are synthesized from aluminosilicate precursors, such as fly ash, Ground Granulated Blast Furnace Slag (GGBS), and metakaolin, using a process called geopolymerization that involves alkaline activation. The geopolymerization process consists of three stages: dissolution, orientation, and poly-condensation [7]. Geopolymer composites have been consistently investigated as potential substitutes for conventional Portland cement-based construction products. Originally, geopolymer study was restricted to the addition of only natural raw materials like kaolin, metakaolin, silica fumes, and calcined clays; however, research has also been expanded to industrial waste products such as palm oil fuel ash, clay-based slag, fly ash, etc., to make it more sustainable regarding availability, the environment, and the economy [8]. According to Khatib et al. [9], environmental sustainability stems from the fact that almost all the precursor materials (natural and industrial waste by-products) have much lower CO_2_ emission factors compared to PC. The high generation (compared to utilization) of industrial waste by-products, disposal issues, and their detrimental/hazardous nature make their immobilization/utilization as a precursor even more environmentally viable. Generally, geopolymer precursor materials, both in their natural and by-product state, are expected to have significant alumina (Al_2_O_3_) and silica (SiO_2_) contents. These aluminosilicate precursors require alkaline activating solutions using sodium- and potassium-based hydroxide (NaOH and KOH) and silicate (Na_2_SiO_3_ and K_2_SiO_3_) mixtures in specified combinations [10]. This was corroborated by Davidovits [11] and suggests that the success of geopolymerization depends on the presence of reactive silica and alumina sources that interact with alkaline activators (potassium or sodium) to create the geopolymeric binder.

Recent studies highlight how alkali-activated slag cement (AAS) offers a promising, sustainable alternative to traditional ordinary Portland cement (OPC). Palomo et al. [12] discovered that AAS achieved a compressive strength of 20–30% higher than OPC after 28 days of curing using fly ash as the main precursor. Meanwhile, Lee and van Deventer [13] developed a geopolymer concrete using fly ash–kaolin formulations as the precursor and highlighted its enhanced resistance to sulphate and acid attacks. Additionally, life-cycle assessments revealed that AAS can reduce CO₂ emissions by 40–70%, as reported by Habert et al. [14]. Incorporating alkali-activated slag (AAS) in concrete mixtures can lower binder costs by 20–30%, primarily due to the use of industrial by-products like slag and fly ash, as noted by Davidovits [15]. These examples illustrate how AAS not only matches but often surpasses OPC in terms of strength, durability, and sustainability.

Due to high chemical activation and processes involving a wide range of parameters, the development of a standard mix design code is complex and still in its elementary stage. Most of the design studies are based on critical parameters that primarily affect its compressive strength and workability/setting to some extent. A regression analysis based on experiments on the simulation of the strength versus water-to-cement ratio curve of normal concrete, the absolute volume method, and the combined grading concept using fly ash–GGBS-activated geopolymer concrete indicated some positive potential [16,17,18,19]. Therefore, this research aims to develop a geopolymer concrete by optimizing geopolymer concrete mixtures with various ratios of Ground Granulated Blast Furnace Slag (GGBS) and pulverized fly ash (PFA) as precursors, which are activated using a laboratory-synthesized silica fume (SF)-derived sodium silicate solution. As such, silica fume containing 85–95% silicon dioxide (SiO_2_) is employed, as it is an effective silica source due to its composition and properties. The findings of this research are expected to provide valuable insights for the construction industry by adding to/creating an understanding of the technical aspects and feasibility of geopolymer concrete. The goal is to contribute to the widespread adoption of geopolymers as a sustainable alternative, paving the way for an environmentally friendly construction future.

## 2. Materials

The materials used in this study include Ground Granulated Blast Furnace Slag (GGBS), pulverized fly ash (PFA), silica fume (SF) sand, coarse aggregates, and sodium hydroxide. The ordinary Portland cement (OPC) utilized in this study is in accordance with BS EN 197-1:2011 [20]. Ground Granulated Blast Furnace Slag (GGBFS) is a by-product of iron and steel manufacturing, created by rapidly cooling molten iron slag from a blast furnace with water or steam. For this research, Class F fly ash was utilized. This is a by-product of thermal power plants generated from pulverized coal combustion in furnaces. The SF used was a commercial reactive micro-silica in the form of a light grey amorphous powder with a silicon dioxide content (SiO_2_) of 97.1%. It was manufactured in Norway by Elkem Silicon Materials and supplied under the trading name of Elkem Undensified Microsilica 971 by Tarmac Cement and Lime Company, Buxton Lime and Powders, Derbyshire, Derby, UK.

Table 1 and Table 2 show the chemical composition and physical properties of the constituent materials used in this study.

This study utilized sand as the fine aggregate, while two sizes of coarse aggregates, 20 mm and 10 mm, were used, in accordance with BS EN 12620:2002+A1:2008 [25].

## 3. Methodology

Geopolymer concrete is produced by activating aluminosilicate-rich materials with alkaline solutions, forming a geopolymer binder through polymerization. Unlike traditional Portland cement, which develops a low crystalline/gel-like formation (calcium silicate hydrate—C-S-H) after hydration, GPC features a microstructure with cross-linked Si-O-Al bonds, providing high strength and durability without calcium in the primary structure.

### 3.1. Mix Design

Geopolymer concrete mixtures were carefully designed to assess the impact of various mix variables on their performance characteristics, including the proportion of Ground Granulated Blast Furnace Slag (GGBS) and pulverized fly ash (PFA), the quantities of alkaline activator solutions, and the ratio of sodium silicate to sodium hydroxide (SS/SH). Approximately ninety trial mixes involving cubes, cylinders, and beams were prepared to evaluate their effects on compressive, tensile, and flexural strength and workability. The alkaline activator employed was a 1:1 blend of sodium silicate (SSA) and sodium hydroxide (SH), with an alkali-to-precursor (A/P) ratio of 0.4 and a water-to-binder (W/B) ratio of 0.55, as advised by Adeleke et al. [26]. For comparative purposes, a control mix of 100% Portland cement is noted as C in this study. The geopolymer mix designs varied in binder proportions as follows: CL0 (50% GGBS—50% PFA), CL1 (60% GGBS—40% PFA), CL2 (70% GGBS—30% PFA), CL3 (80% GGBS—20% PFA), and CL4 (90% GGBS—10% PFA). This approach aimed to provide comprehensive insights into optimizing the precursor blends (GGBS-PFA) for improved mechanical performance and workability while advancing sustainable construction practices.

### 3.2. Preparation of Alkaline Activator

The alkaline activator was created by combining the sodium hydroxide solution with the sodium silicate solution just before mixing to ensure the solution’s reactivity. Firstly, the alkaline activator (sodium hydroxide solution) was prepared in the laboratory by dissolving laboratory-grade NaOH pellets (98% purity) from Fisher Scientific UK in deionized water, as outlined by Adeleke et al. [26] using Equation (1).


NaOH(s) + H_2_O(l) → NaOH(l) (1)

When water was added to NaOH pellets, a percentage of heat was generated due to the exothermic reaction. In this research, the alkaline activator was a 10 M sodium hydroxide (NaOH) solution with a density of 1.33 g/cm^3^. The molar weight of NaOH is 40 g/mol. Therefore, to prepare a 10 molar (10 M) solution, 400 g of NaOH pellets should be dissolved in water to make a total volume of 1 L.

Secondly, incorporating sodium silicate improves the formation of geopolymer precursors and accelerates the polymerization process [27]. Sodium silicate alternative (SSA) was prepared by combining SF with the previously prepared sodium hydroxide solution to create SSA at specified mix proportions using Equation (2). The solution was stirred for 4 to 5 min to ensure the thorough mixing of SF and NaOH to form the SSA (Na₂SiO₃), which must be stored at room temperature for 24 h prior to use in the overall concrete mix. Additionally, the relative humidity should be maintained at around 65% before using it for casting [26,28].


2SiO_2_ + 2NaOH(s) → NaO(SiO_2_)_2_(l) + H_2_O(l) (2)

### 3.3. Preparation of Traditional and Geopolymer Concrete Specimens

An optimized mixing procedure was systematically employed to prepare the geopolymer concrete, ensuring uniformity and consistency throughout the mixture. Initially, the dry ingredients comprising PC, the precursors (GGBS and PFA), and both fine and coarse aggregates were precisely weighed according to the specifications outlined in Table 3. The aggregates and source materials were thoroughly dry-mixed and placed in a rotating pan mixer with a capacity of 113 L (163 kg) for approximately two minutes to ensure a homogeneous blend. For the traditional concrete, appropriate materials were measured and mixed according to the mix design. Subsequently, a carefully measured amount of alkaline solution according to the mix design was slowly added to the dry ingredients and mixed for two minutes to ensure a homogeneous geopolymer concrete mixture. After that, the required water was added, and the mixture was then subjected to a final two-minute mixing phase to achieve the desired consistency of the fresh concrete.

### 3.4. Casting and Curing Procedures

The fresh concrete was cast into cubical, cylindrical, and rectangular moulds immediately after mixing according to BS EN 12390-1:2021 [29], while concrete compaction was achieved using a vibration table in accordance with BS EN 12390-2:2019 [30]. Twelve cubes (100 mm × 100 mm × 100 mm), two cylindrical specimens (100 mm diameter × 200 mm height), and two beams (100 mm × 100 mm × 500 mm) were cast for each mix composition [31]. The test samples were removed from their moulds after 24 h and then subjected to moist curing in a container for 3, 7, 28, and 56 days at room temperature according to BS EN 12390-2:2019 [30].

### 3.5. Geopolymer Concrete Specimen Testing Method

The consistency of the fresh concrete was evaluated using the slump test in accordance with BS EN 12350-2:2019 [32]. The hardened characteristics of the concrete were investigated using unconfined compressive strength (UCS) at curing intervals of 3, 7, 28, and 56 days, while the tensile splitting strength (TSS) and flexural strength were measured at 28 days. The volumetric density of the geopolymer concrete was determined based on BS EN 12390-7:2019 [33]. Moreover, density tests were conducted according to BS EN 12390-4:2019 [34] prior to UCS testing, which involved crushing concrete cubes to failure at a rate of 6 kN/s. In addition, concrete cylinders underwent a 28-day tensile splitting strength test according to BS EN 12390-6:2019 [35], while the flexural strength of concrete beams was tested after 28 days using the Avery Denison machine, with a loading rate of 10 kN/min according to BS EN 12390-5:2019 [36]. Six beams (100 mm × 100 mm × 500 mm) were tested under two concentrated loads spaced 100 mm apart (4-point loading system).

## 4. Results and Discussion

### 4.1. Slump Test

**Figure 1:**
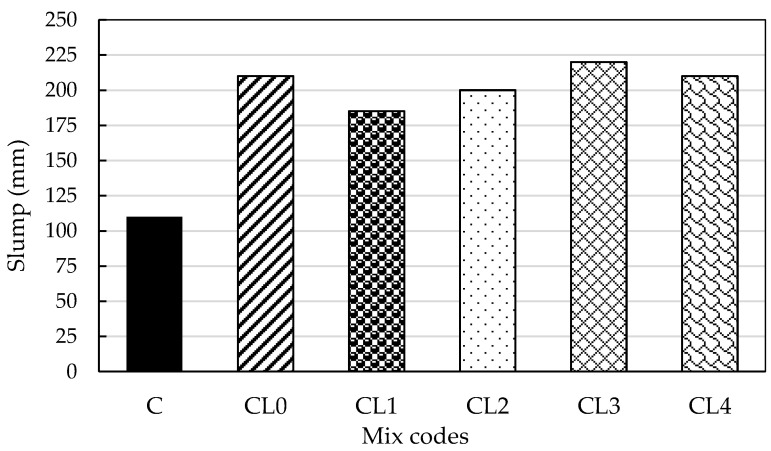
Slump data for fresh OPC and geopolymer concrete mixes.

The slump test, a fundamental evaluation method in concrete technology, is used to evaluate the workability of freshly mixed concrete. Figure 1 presents the measured slump values for various concrete mixes, which were conducted to assess the consistency of the fresh concrete. Observations showed that the control mix (C), identified as 100% PC, demonstrated the lowest slump value of 110 mm. The geopolymer concrete mix CL1 recorded the lowest slump value of 185 mm, whereas the highest slump value of 220 mm was registered for mix CL3. The data indicate that consistency is generally enhanced by keeping the A/P ratio constant while changing the content of the GGBS and PFA.

With an activator-to-precursor (A/P) ratio of 0.4, the workability of the geopolymer concrete is evaluated across various mix proportions of Ground Granulated Blast Furnace Slag (GGBS) and pulverized fly ash (PFA). This ratio ensures there is enough alkaline activator for the geopolymerization process while keeping the mix workable, avoiding excessive liquidity or stiffness. Rangan et al. [37] found that this A/P ratio achieves optimal mechanical strength and workability, enhancing flowability significantly compared to conventional cement mixes. The combination of GGBS and PFA demonstrates how their unique properties synergize to improve workability, making geopolymer concrete a compelling option for applications needing higher workability without sacrificing strength.

### 4.2. Density of Geopolymer Concrete

**Figure 2:**
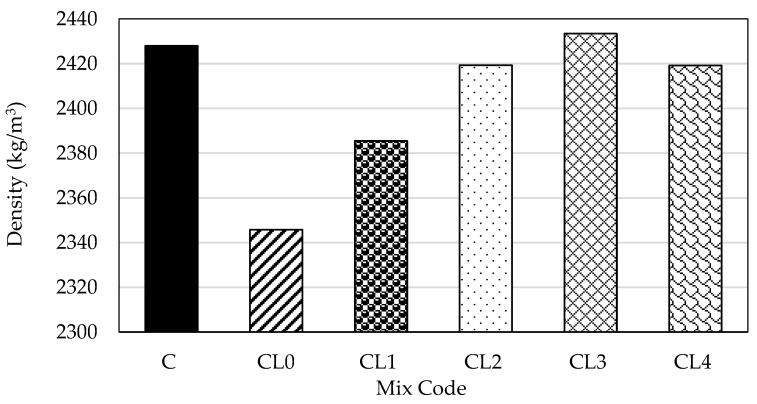
Mean density of hardened concrete.

Figure 2 shows the average density data of the hardened concrete samples. The specimen CL3 achieved the highest density value, while mix CL0 attained the lowest density value of 2346 kg/m^3^. Observations show a progressive increase in density strength values with every percentage increase in PFA except for mix CL4, which could be suggested as an anomaly. The minor differences in these densities may be attributed to slight variations in mix proportions or curing conditions. Nevertheless, the results generally suggest a uniform and well-compacted material.

### 4.3. Compressive Strength of Hardened Geopolymer Concrete

**Figure 3:**
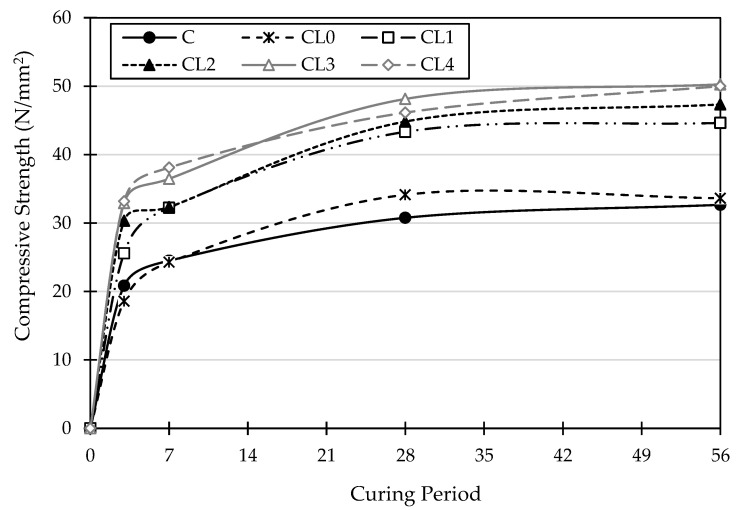
Compressive strength results at various curing times of concrete mixtures.

Figure 3 shows the compressive strength of the concrete specimens evaluated over 56 days of curing. The data revealed a clear trend of increasing compressive strength with curing age across all mix designs, highlighting the beneficial effects of extended curing. While the control mix (C) demonstrated a steady growth from 20.83 N/mm^2^ at 3 days to 32.67 N/mm^2^ at 56 days, the geopolymer concrete mixes displayed varying rates of strength development depending on their composition. Regarding the geopolymer concrete, CL0 displayed the lowest strength but was slightly increased compared to the control mix (C) over the curing period. In addition, mix CL3 displayed the highest compressive strength, surpassing the control mix (C) by approximately 54%. The data reveal a clear trend across all geopolymer concrete mixtures, indicating that compressive strength generally improves as the GGBS content increases and the PFA content decreases, provided the A/P ratio is maintained at 0.4. This is attributed to the ongoing hydration and polymerization reactions within the geopolymer matrix, leading to the formation of a denser and stronger microstructure. Adding GGBS enhances particle packing, reducing voids and strengthening the matrix. Provis and Bernal [38] note that the GGBS calcium content speeds up hydration, forming a dense network of C-A-S-H and N-A-S-H gels. Xu et al. [27] further highlight that GGBS refines the pore structure, significantly boosting compressive strength over time. The findings of this study highlight the crucial role of mix design in influencing the compressive strength development of geopolymer concrete. By precisely adjusting the proportions of raw materials, desired strength levels can be achieved at various curing ages.

Adam [39] observed that strength increased with a higher activator-to-binder ratio but declined significantly beyond a certain point, with a ratio of 0.55 being optimal. This finding was also confirmed by Al Bakri Abdullah et al. [40] and Liyana et al. [41]. By using this W/B ratio, the concrete achieves strong compressive strength and meets density requirements, like traditional concrete. Additionally, it enhances durability, reduces permeability, and improves resistance to harsh environments, making it a sustainable alternative to Portland cement-based concrete.

Observations showed that increasing the content of Ground Granulated Blast Furnace Slag (GGBS) while decreasing pulverized fly ash (PFA) significantly enhances compressive strength. Although PFA contributes to strength, GGBS has a more pronounced effect, with compressive strength improving over time in various mixtures. The strength gain in geopolymer concrete mixes results from the pozzolanic reaction between supplementary materials and alkaline activators, forming aluminosilicate gels (N-A-S-H), crucial for strength development [42]. The increase in compressive strength with higher GGBS content is due to both physical and chemical factors. Provis et al. [38] highlight that GGBS improves particle packing, creating a denser microstructure with less porosity. Nath and Sarker [16] add that the calcium content of GGBS accelerates the formation of hydration products like C-A-S-H and C-S-H gels, which strengthen the matrix. Together, these effects enhance the mechanical properties of geopolymer concrete. Smith et al. [43] showed that adding supplementary cementitious materials like fly ash or silica fume to concrete mixtures notably improves compressive strength, especially during the later stages of curing. Among the mixtures, CL3 exhibits the highest compressive strength, which is approximately 54% higher than the control mix (C). However, Chowdhury et al. [44] found that further increases beyond a certain level led to a decline in strength. Optimizing mixture composition, as in CL3, enhances strength, and future research should address balancing modifications with workability to avoid plateaus like in CL4.

### 4.4. Split Tensile Strength

**Figure 4:**
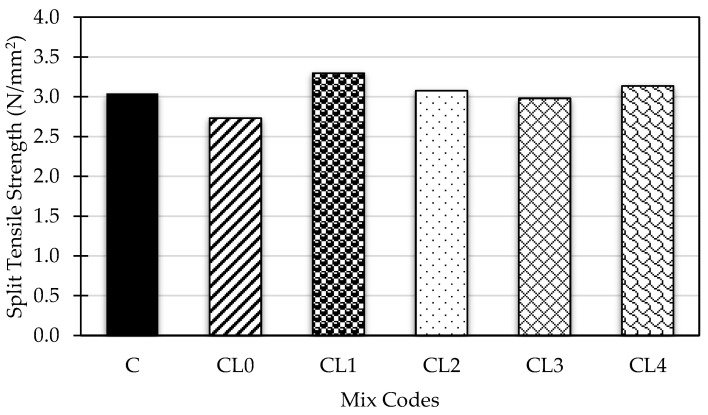
Mean split tensile strength (N/mm^2^) of concrete mixtures after 28 days of curing.

This section outlines the process for determining the average split tensile strength of all concrete specimens after 28 days of production. The specimens were moist-cured under ambient room temperature. The mean split tensile strength values for the concrete mixes are presented in Figure 4.

A notable difference in tensile splitting strength was observed between the OPC control sample and the GGBS-PFA-based geopolymer samples. The control mix provides the baseline for comparison, which has a split tensile strength of 3.04 N/mm^2^. In comparison, CL1 demonstrates the highest split tensile strength with 3.31 N/mm^2^, suggesting that the mix design at this level optimally enhances the tensile properties of the geopolymer concrete. CL0 and CL3, having a value of 2.73 N/mm^2^ and 2.98 N/mm^2^, respectively, show a slight decrease compared to the control mix. The mixtures of CL2 and CL4 exhibit more significant enhancements, likely due to more effective alterations in the mix design, with recorded tensile strength values of 3.08 N/mm^2^ and 3.14 N/mm^2^ after 28 days of curing time.

The tensile strength results for the geopolymer concrete mixtures designed with an alkali-to-precursor (A/P) ratio of 0.40 and a water-to-binder (W/B) ratio of 0.55 reveal significant trends in the impact of varying proportions of Ground Granulated Blast Furnace Slag (GGBS) and pulverized fly ash (PFA) on mechanical performance. These ratios create an ideal environment for activating GGBS, resulting in tensile strengths competitive with traditional cement-based concrete. Increasing the GGBS content improves tensile strength up to 60% (CL1), beyond which strength stabilizes or slightly decreases. A study by T.L. Prasad et al. [45] revealed that an increasing GGBS content in geopolymer concrete improved tensile and flexural strength up to an optimal level, after which the strength levelled off or showed minimal reduction. A moderate PFA content (10–30%) paired with higher GGBS still achieves comparable or superior performance, indicating that optimized geopolymer concrete mixtures can serve as a sustainable alternative for construction applications.

### 4.5. Flexural Strength

**Figure 5:**
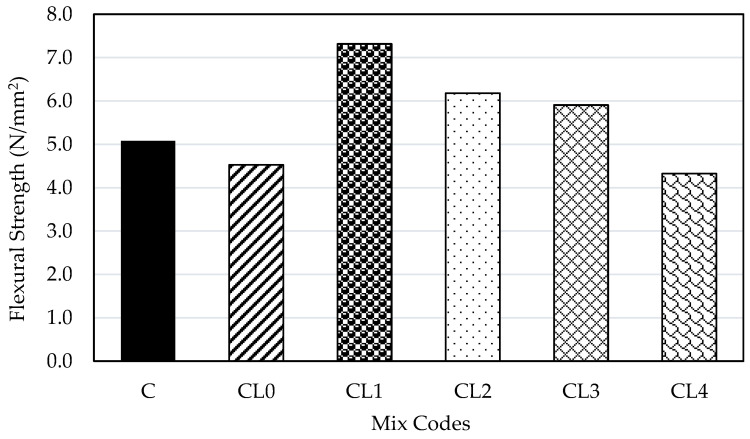
Flexural strength of concrete specimen after 28 days of curing.

Figure 5 displays the flexural strength results for the concrete specimens cured at ambient temperature and assessed 28 days after casting. The beams were tested by the two-point loading method in the Avery Denison flexural machine. Based on the flexural test, the control mix exhibited a flexural strength of 5.08 N/mm^2^. Geopolymer mixes CL0, CL1, CL2, CL3, and CL4 show varied flexural strength values, with CL1 achieving the highest strength of 7.32 N/mm^2^, exceeding the control mix by 45%. CL2 and CL3 also outperform the control mix, with flexural strengths of 6.18 N/mm^2^ and 5.91 N/mm^2^, respectively. However, CL0 and CL4 exhibit lower flexural strengths compared to the control mix, with values of 4.53 N/mm^2^ and 4.33 N/mm^2^, respectively.

The flexural strength of geopolymer concrete varies significantly compared to the control mix (C). The CL1 geopolymer mix demonstrates the highest flexural strength, which is 44% greater than the baseline. This indicates an optimal balance between GGBS and PFA, enhancing resistance to bending stresses. Conversely, mixes with excessive GGBS, like CL4, show a decrease in flexural strength, dropping to 41% of CL1. This study highlights the importance of optimizing the blend of GGBS and PFA to achieve the best mechanical properties in geopolymer concrete, with CL1 showing the most promising performance for practical applications. In the geopolymer concrete mix CL0, cube and cylinder specimens showed inadequate hardening within 24 h, requiring 2–3 days before demoulding under ambient curing. A hairline crack was observed on the beam specimen, though strong adhesion to the steel mould was noted due to its dense microstructure and the formation of C-A-S-H and N-A-S-H gels [38]. Nath and Sarker [22] noted that including GGBS in fly ash-based geopolymer concrete improves setting and early strength properties, contributing to the strong bond. Further investigation of the CL0 mix design and curing conditions is needed to resolve early hardening issues. Studying curing methods, microstructure, and reinforcement can improve adhesion, prevent cracking, and enhance geopolymer concrete performance.

## 5. Conclusions

The outcome of this study suggests the practicality of developing a PFA-GGBS-based precursor for geopolymer concrete production as a sustainable alternative to Portland cement in concrete production. Therefore, the following conclusions of this study are highlighted below:

1. The geopolymer concrete cured under ambient conditions achieved a mean compressive strength ranging from 27 N/mm^2^ to 42 N/mm^2^, with strength increasing as the GGBS content increased from CL0 to CL4. This shows the positive impact of a large GGBS content in the PFA-GGBS mix regime.
2. All geopolymer mixes generally showed higher compressive strengths than the control mix. However, the split tensile strength did not increase proportionally, which may need consideration for tensile-critical applications.
3. The geopolymer concrete with an A/P ratio of 0.4 and a W/B ratio of 0.55 showed excellent workability, strength, and density, making it suitable for practical use.
4. Despite the positive outcomes of developing the PFA-GGB precursor-based geopolymer formulations, careful consideration and strategies such as proper material storage (activators and precursor) under appropriate conditions, machine mixing only, and ready-mix concrete should be adopted. This replicates the laboratory mixing programme, and batch mixing operators must have adequate knowledge of geopolymer concrete for it to be considered and adopted for large-scale production on a construction site.
5. Generally, the drive to move to geopolymer concrete is expected to reduce the need for Portland cement, which will positively impact the drive for sustainability in the construction industry.

## Figures and Tables

**Table 1:** Chemical composition (%) of OPC [21], GGBS [21], PFA [22,23], and SF [24].

Constituents (wt%)	OPC	GGBS	PFA	SF
CaO	61.49	37.99	2.27	0.2
MgO	3.54	8.78	1.05	0.1
SiO_2_	18.84	35.54	62.2	97.1
Al_2_O_3_	4.77	11.46	27.5	0.2
Na_2_O	0.02	0.37	0.52	-
P_2_O_5_	0.1	0.02	-	0.03
Fe_2_O_3_	2.87	0.42	0.56	0.01
Mn_2_O_3_	0.05	0.43	-	-
K_2_O	0.57	0.43	0.86	0.2
TiO_2_	0.26	0.7	1.54	-
V_2_O_5_	0.06	0.04	-	-
BaO	0.05	0.09	-	-
SO_3^−^_	3.12	1.54	0.38	0.1
Loss on ignition	4.3	2	0.64	0.5

**Table 2:** Physical properties of the coarse and fine aggregates [21].

Property	Fine Aggregates (Sand)	Coarse Aggregates	
		10 mm	20 mm
Uniformity coefficient (CU)	0.11	3.3	1.3
Curvature coefficient (CC)	1.75	1.5	7.5
Flakiness index (%)	-	30–35	23
Elongation index (%)	-	17–22	12
Shape index (%)	-	12	7
Impact value		23	15
Fineness modulus (mm)	1.54	4	-
Uncompacted bulk density (g/cm^3^)	1.5	1.35	2.57
Pre-dried particle density (g/cm^3^)	2.6	2.69	-
Water absorption (%)	21	2	1.1

**Table 3:** Mix proportions for ordinary and geopolymer concrete production.

Mix Code	Elaborated Abbreviation	Concrete Binder							Water (L)	Aggregates (kg)		
		PC (kg)	Geopolymer							Fine Agg.	Coarse Agg.	
			GGBS (kg)	PFA (kg)	A/P Ratio	SSA:SH	Activator (mL)					
							SSA	SH			10 mm	**20 mm**
C	OPC (Control 1)	8.9	-	-	-	-	-	-	4.9	17.7	8.8	17.8
CL0	CL0—50% GGBS—50% PFA	-	3.2	3.2	0.4	01:01	865	865	3.1	17.7	8.8	17.8
CL1	CL1—60% GGBS—40% PFA	-	3.8	2.5	0.4	01:01	865	865	3.1	17.7	8.8	17.8
CL2	CL2—70% GGBS—30% PFA	-	4.4	1.9	0.4	01:01	865	865	3.1	17.7	8.8	17.8
CL3	CL3—80% GGBS—20% PFA	-	5.1	1.3	0.4	01:01	865	865	3.1	17.7	8.8	17.8
CL4	CL4—90% GGBS—10% PFA	-	5.7	0.6	0.4	01:01	865	865	3.1	17.7	8.8	17.8
OPC—ordinary Portland cement, GGBS—Ground Granulated Blast Furnace Slag; A/P—activator/precursor ratio, SSA:SH—sodium silicate alternative to sodium hydroxide ratio, PFA—pulverised fly ash; W—Water.												

## Data Availability

The original contributions presented in this study are included in the article. Further inquiries can be directed to the corresponding author.
